# Effects of rosemary extract and its residue on production, immune performance, and gut microbiota in geese

**DOI:** 10.3389/fmicb.2024.1483626

**Published:** 2025-01-08

**Authors:** Yuzhi Huang, Lanmeng Xu, Hang He, Lijuan Peng, Qinfeng Liao, Kun Wan, Simeng Qin, Lijing Cao, Jie Zhang

**Affiliations:** ^1^College of Animal Science and Technology, Southwest University, Chongqing, China; ^2^College of Animal Science and Technology, Chongqing Three Gorges Vocational College, Chongqing, China; ^3^Chongqing Rongchang District Vocational Education Center, Chongqing, China

**Keywords:** rosemary extract, rosemary extract residue, production performance, immune performance, gut microbiota, geese

## Abstract

**Introduction:**

To explore the effects of rosemary extract (RE) and its residue (RR) on the production, immune performance, and gut microbiota of geese.

**Methods:**

We treat 28-day-old Sichuan white geese (*n* = 180) with three diets: (1) basal diet (control), (2) basal diet supplemented with 0.02% RE, and (3) basal diet supplemented with 15% RR for 42 days.

**Results and discussion:**

On day 70, compared with control treatment, the final body weight, average daily gain and lysozyme levels in the RE treatment increased significantly (*p* < 0.05). In the RE and RR treatments, there was a significant decrease in alkaline phosphatase, globulin, and high-density lipoprotein levels compared to the control treatment, and there was also a significant increase in aspartate aminotransferase/alanine aminotransferase (*p* < 0.05). Moreover, for both RE and RR treatments, semi-eviscerated, eviscerated weights, and calcium apparent digestibility increased significantly, along with a decrease in the duodenal index (*p* < 0.05). Compared with RE treatment, those in the RR treatment had significantly higher duodenal and jejunum relative lengths, aspartate aminotransferase, uric acid, total cholesterol, and low-density lipoprotein levels, and decreased chest depth, chest angle, neck length, semi-eviscerated and eviscerated weights, crude protein digestibility, and levels of globulin, triglyceride, and lysozyme (*p* < 0.05). There were no differences in gut microbiota α or β diversities among treatments (*p* > 0.05). Compared to the control treatment, the relative abundance of *Turicibacter* significantly increased in the RR and RE treatments, and the relative abundance of *Sporobacter, Alistipes*, and *Barnesiella* significantly increased in the RR treatment (*p* < 0.05). Rikenellaceae, Succinivibrionaceae, and Aeromonadales were enriched in the RR treatment, and Lachnospiraceae, Turicibacteraceae, Fusobacteriaceae, and Enterobacteriaceae were enriched in the RE treatment. While we demonstrate the RR diet to be less effective than the RE diet, it did improve production and the gut microbiota of geese to a certain extent.

## 1 Introduction

Rosemary (*Rosmarinus officinalis*), a perennial plant in the family Lamiaceae, native to the Mediterranean region, has been widely cultivated in southwest China (González-Trujano et al., 2007). It is now widely used in cosmetics, pharmaceuticals, and food additives, as well as for cancer prevention (Choi et al., 2019). Rosemary contains active ingredients, mostly comprising polyphenols, flavonoids, alkaloid, glycoside, and terpenoids, among which carnosol, and carnosic and rosmarinic acids are typical. These compounds are known for their anti-microbial, anti-inflammatory, immune-boosting, and growth-promoting functions (Rodrigo et al., 2022; Sanchez-Camargo and Herrero, 2017). Many studies have demonstrated that rosemary extract (RE) positively affects animal production performance. Adding 100 mg.kg^−1^ of RE to the diet of broiler chickens can improve their average daily gain (ADG) and inhibit *Escherichia coli, Salmonella indiana*, and *Listeria innocua* activities to maintain intestinal homeostasis (Mathlouthi et al., 2012); adding 250–750 g.t^−1^ of RE to the diet can improve immune indicators such as IgG and IgM levels, as well as feed-use efficiency (Yao et al., 2023); adding 10 g.kg^−1^ of rosemary leaf powder to the diet of tilapia significantly increased levels of lysozyme and complement (Naiel et al., 2020); and adding 1,000 ppm rosemary extract significantly reduced the microbial count in pork and extended its shelf life (Peñaranda et al., 2021).

The current yield of rosemary essential oil obtained through distillation is relatively low. Moreover, there is a significant amount of solid residue generated annually, ranging from 10 to 20 × 10^3^ tons (Oreopoulou et al., 2021). The rosemary residue (RR) after extraction of these active ingredients still retains a considerable amount of carnosol, carnosic, and rosmarinic acids (Ayyildiz et al., 2024). This solid residue is an underutilized waste, which is a significant oversight given its potential to contain valuable bioactive ingredients that could be utilized for various applications. Few studies have reported the effects of adding RR to animal diet. For example, its addition to lamb feed can increase polyunsaturated fatty acid contents and its ratio to saturated fatty acids, and the vitamin E content of muscle, which in turn can lead to an increase in ADG and a reduction in feed conversion ratio (FCR; Smeti et al., 2021; Yagoubi et al., 2021, 2018). In summary, it is unknown if addition of RE or RR further affects performance such as goose production and immunity, as well as gut microbiota.

China leads the globe in goose production, hitting an annual output of 4.29 million tons (Zhang Y. et al., 2023). However, an antibiotic ban coupled with rise in feed prices has increased costs of raising these birds. Identifying natural plant ingredients that can improve animal growth performance is a priority. Based on the doses of RE and RR used in previous studies (Lee et al., 2018; Smeti et al., 2021), as well as the result of our small-scale exploratory experiment. We investigate the effects of adding 0.02% RE or 15% RR to the diet on growth, carcass, and immune performance, serum metabolites, and gut microbiota of geese. The substantial amount of rosemary residues that retain bioactive ingredients from the industrial extraction process offers the potential to serve as an inexpensive feed resource in meat goose production.

## 2 Materials and methods

### 2.1 Animals, diets, and experimental design

After drying the leaves of rosemary, they were crushed and distilled with water vapor for 1.5 h to obtain the extract. The extract was then subjected to rotary evaporation concentration and drying treatment to obtain RE powder. The contents of carnosic acid, carnosol, and rosmarinic acid were 22.76%, 16.23%, and 5.17%, respectively. In addition, the remaining residue after extracting rosemary leaves was dried and crushed, with contents of carnosic acid, carnosol, and rosmarinic acid at 0.46%, 1.55%, and 0.35%, respectively.

A total of 180 healthy Sichuan white geese (mixed sex) at 28-day-old age having an average body weight of 0.95 ± 0.02 kg were used and randomly divided into three groups, six replicates in each group and 10 geese in each replicate. The control (Con) group was fed with basal diet, while the treatment group added 15% RR or 0.02% RE to the basal diet, respectively. Feed was provided three times daily at 7:00, 12:00, and 18:00 h. Geese were allowed access to feed (in pellet form) and water *ad libitum* throughout the 42 days of the experiment. Adopting conventional feeding management and immunization procedures, natural lighting, and ventilation. The corn-soybean based basal diet was formulated to be fed from day 28 to 70 (Table 1). All animal works were conducted according to the guidelines for the care and use of experimental animals established by the Ministry of Agriculture and Rural Affairs of China. Animal Care and Use Committee in Southwest University approved this project (SWU-20143003). The study was conducted in accordance with the local legislation and institutional requirements.

**Table 1:** Composition and nutrient level of basal diet (air-dry basis).

**Ingredients**	**Content (%)**	**Nutrient**	**Content**
Corn	53.60	Metabolizable energy (MJ/kg)^b^	11.21
Soybean meal	11.50	Crude protein (%)	14.81
Wheat bran	14.50	Crude fiber (%)	8.04
Rice bran	13.40	Calcium (%)	0.80
Silkworm chrysalis	1.79	Available P (%)^b^	0.40
CaHPO_4_	0.90	Lysine (%)	0.85
Salt (NaCl)	0.20	Methionine (%)	0.30
Limestone	0.75		
L-Lysine (98%)	0.18		
DL-Methionine	0.07		
Choline chloride	0.12		
Premix^a^	2.00		
Sand	1.00		
Total	100		

^a^Premix contained the following per kg of diet: VA 40,000 IU, VD_3_ 2,000 IU, VE 60 mg, VK_3_ 2 mg, VB_1_ 4 mg, VB_2_ 24 mg, VB_6_ 4 mg, VB_12_ 50 μg, nicotinic acid 12 mg, pantothenic acid 36 mg, folic acid 4 mg, biotin 0.4 mg, Fe 120 mg, Cu 4 mg, Mn 300 mg, Zn 160 mg, I 0.2 mg, and Se 0.2 mg.

^b^ME was a calculated value, while the others were measured values.

### 2.2 Sampling and analyses

#### 2.2.1 Growth performance

Feed consumption before the feeding and mortality was recorded during the experiment. Body weight was measured on day 70 with empty stomach. ADG, average daily feed intake (ADFI), and FCR were calculated.

#### 2.2.2 Body size

The body size including body slope length, chest width, chest depth, chest girth, keel length, shank length, pelvis width, chest angle, neck length, and shank girth were determined using caliper or tape according to the China national standard of NY/T 823-2020.

#### 2.2.3 Serum metabolites

Blood of fasted geese were collected from vena brachialis under the wing, and then centrifugation at 3,500 × g for 10 min to obtained serum. Serum aspartate aminotransferase (AST), alanine aminotransferase (ALT), alkaline phosphatase (ALP), total protein (TP), albumin (ALB), globulin (GLO), uric acid (UA), total cholesterol (TC), triglycerides (TG), high-density lipoprotein (HDL), low-density lipoprotein (LDL), IgG, IgM, IgA, and lysozyme levels were measured using a clinical chemical analyzer (CL-8000, Shimadzu, Japan) or commercial kits (Solarbio, Beijing, China).

#### 2.2.4 Carcass and organ index

After slaughter, the feathers, foot cuticle, toe shell, and beak shell were manually removed immediately after scalding at 60°C for ~2 min. Next, the eviscerated, half-eviscerated, leg muscle, breast muscle, spleen, liver, pancreas, gizzard, glandular, duodenal, jejunum, and ileum were removed and weighed, and calculated the organ index = (organ weight/BW) × 100%. Furthermore, the relative intestinal length = intestinal length/BW.

#### 2.2.5 Nutrient digestibility

At day 64, a healthy goose close to the average BW of the replicate was selected, moved to the metabolic cage for pre feeding for 3 days, and recorded the feed intake at day 67 to 69. Total excreta were collected and weighed after removing the feathers and other debris. Excreta were drying in 65°C for 48 h, and then crushed and passed through a 40 mesh sieve. Crude protein, Ca, and P were determined according to the China national standards of GB/T 6432-2018, GB/T 6436-2018, and GB/T 6437-2018 using Kjeldahl nitrogen, EDTA complex titration, and spectrophotometry method, respectively. Total energy was determined by the combustion method.

#### 2.2.6 Gut microbiota

Cecal contents were collected and put into sterile cryotubes, quick-frozen with liquid nitrogen, and −80°C storage. Bacterial genomic DNA was extracted using the DNA extraction kit (Qiagen, Germany) following the manufacturer's protocol. DNA was amplified using primers (319 F: 5′-ACTCCTACGGGAGGCAGCAG-3′; 806R: 5′-GGACTACHVGGGTWTCTAAT-3′) specific for the V3–V4 region of the 16S rRNA gene, and the constructed library was paired-end sequenced by Illumina HiSeq 2500 (Illumina, USA). After a series of processing such as concatenation, screening, quality control, and filtering of the raw data, the USEARCH was used to cluster and generate operational operation units (OTUs) based on 97% similarity. The OTUs were blasted with the Silva (https://www.arb-silva.de/) database using RDP Classifer for species annotation, with a confidence threshold of 0.6. The diversity, composition, and taxa enrichment of the microbial communities were further analyzed using mothur, QIIME2 (https://qiime2.org/) and linear discriminant analysis effect size (LEfSe), respectively.

### 2.3 Statistical analysis

The data analyses were performed with SPSS 22.0 (IBM, USA) using one-way ANOVA with Duncan's multiple comparison tests. Values are expressed as means ± standard deviation (SD), and a probability level of *p* < 0.05 was considered to be statistically significant.

## 3 Results

### 3.1 Growth performance

Compared with Con treatment, the final body weight and ADG of geese in the RE treatment increased significantly (*p* < 0.05), while there was no significant change in ADG in RR treatment (*p* > 0.05; Table 2).

**Table 2:** Effect of RR and RE on growth performance of goose.

**Item**	**Group**			***p*-value**
	**Con**	**RR**	**RE**	
Initial body weight (kg)	0.96 ± 0.02	0.95 ± 0.02	0.95 ± 0.02	0.67
Final body weight (kg)	3.10 ± 0.15^b^	3.18 ± 0.32^b^	3.37 ± 0.28^a^	3.03 × 10^−4^
ADG (g)	50.92 ± 3.59^b^	52.96 ± 7.60^b^	57.62 ± 6.81^a^	3 × 10^−4^
ADFI (g)	192.87 ± 13.32	180.47 ± 7.54	180.39 ± 6.73	0.27
FCR	3.79 ± 0.08	3.55 ± 0.66	3.17 ± 0.54	0.38

ADG, average daily gain; ADFI, average daily feed intake; FCR, feed conversion ratio.

^a, b^Mean values within a row bearing different superscripts differ significantly (p < 0.05).

### 3.2 Body size

Compared with Con treatment, chest depth and neck length in RE treatment geese were significantly higher (*p* < 0.05), and values in RE treatment were significantly higher than those for RR treatment (*p* < 0.05; Table 3). Chest angle in RR treatment geese was significantly lower than that in Con and RE treatment (*p* < 0.05).

**Table 3:** Effect of RR and RE on body size of goose.

**Item**	**Group**			***p*-value**
	**Con**	**RR**	**RE**	
Body slope length (cm)	31.01 ± 1.40	31.03 ± 1.54	32.55 ± 2.16	0.12
Chest width (mm)	116.45 ± 10.38	119.24 ± 9.09	116.55 ± 8.24	0.77
Chest depth (mm)	93.80 ± 8.44^b^	95.93 ± 6.62^b^	102.77 ± 5.31^a^	0.029
Chest circumference (cm)	38.78 ± 2.11	37.04 ± 2.49	36.31 ± 2.02	0.07
Sternum length (cm)	16.31 ± 1.18	16.55 ± 0.78	16.18 ± 1.46	0.69
Shank length (mm)	89.46 ± 5.80	91.13 ± 5.92	94.38 ± 6.36	0.23
Hip width (mm)	53.38 ± 7.65	58.90 ± 5.87	58.13 ± 6.79	0.20
Chest angle (°)	64°11′± 9.62^a^	55°12′± 4.29^b^	64°38′± 9.75^a^	0.033
Neck length (cm)	26.06 ± 2.11^b^	24.29 ± 1.25^b^	28.24 ± 2.23^a^	9 × 10^−4^
Shank circumference (cm)	4.49 ± 0.67	4.38 ± 0.35	4.76 ± 0.77	0.43

^a, b^Mean values within a row bearing different superscripts differ significantly (p < 0.05).

### 3.3 Carcass characteristics

Compared with Con treatment, both RE and RR treatment geese had significantly higher semi-eviscerated and eviscerated percentage (*p* < 0.05), and that for RE treatment was significantly higher than that for RR treatment (*p* < 0.05; Table 4).

**Table 4:** Effect of RR and RE on carcass performance of goose.

**Item**	**Group**			***p*-value**
	**Con**	**RR**	**RE**	
Semi-eviscerated (%)	81.84 ± 0.78^c^	82.92 ± 0.42^b^	84.75 ± 0.74^a^	6.27 × 10^−6^
Eviscerated (%)	74.84 ± 0.59^c^	77.00 ± 0.61^b^	78.26 ± 1.35^a^	4.98 × 10^−7^
Breast muscle (%)	9.73 ± 0.34	9.71 ± 0.43	9.74 ± 0.52	0.99
Leg muscle (%)	12.62 ± 0.46	12.47 ± 0.57	12.19 ± 0.34	0.29

^a–*c*^Mean values within a row bearing different superscripts differ significantly (p < 0.05).

### 3.4 Organ index

Compared with Con treatment, the duodenal index of RE and RR treatment geese was significantly lower (*p* < 0.05). The jejunum index, and relative lengths of the duodenum and jejunum were significantly lower in RE treatment (*p* < 0.05), while there were no significant changes in RR treatment (*p* > 0.05; Table 5).

**Table 5:** Effect of RR and RE on organ index of goose.

**Item**	**Group**			***p*-value**
	**Con**	**RR**	**RE**	
Spleen (%)	0.11 ± 0.03	0.12 ± 0.02	0.09 ± 0.02	0.12
Liver (%)	2.08 ± 0.24	1.89 ± 0.30	1.97 ± 0.38	0.56
Pancreas (%)	0.25 ± 0.06	0.21 ± 0.06	0.21 ± 0.04	0.27
Gizzard (%)	2.51 ± 0.36	2.51 ± 0.23	2.35 ± 0.30	0.50
Proventriculus (%)	0.33 ± 0.05	0.32 ± 0.04	0.27 ± 0.05	0.13
Duodenum (%)	0.33 ± 0.04^a^	0.27 ± 0.04^b^	0.24 ± 0.04^b^	0.006
Jejunum (%)	0.75 ± 0.07^a^	0.62 ± 0.19^a^	0.53 ± 0.07^b^	0.02
Ileum (%)	0.46 ± 0.11	0.38 ± 0.08	0.35 ± 0.06	0.09
Duodenum (cm·kg^−1^)	11.49 ± 0.52^a^	11.15 ± 1.00^a^	9.60 ± 0.54^b^	8 × 10^−4^
Jejunum (cm·kg^−1^)	24.87 ± 1.69^a^	24.16 ± 2.20^a^	21.81 ± 1.90^b^	3.8 × 10^−3^
Ileum (cm·kg^−1^)	18.45 ± 0.91	19.26 ± 2.40	18.19 ± 2.75	0.68

^a, b^Mean values within a row bearing different superscripts differ significantly (p < 0.05).

### 3.5 Serum metabolites

Compared with Con treatment, levels of ALP, TP, ALB, and HDL in RR and RE treatment were significantly lower (*p* < 0.05), while ratios of AST/ALT were significantly higher (*p* < 0.05; Table 6). Levels of AST, UA, and LDL in RR treatment were significantly higher (*p* < 0.05), while levels of TG were significantly lower (*p* < 0.05). Levels of AST, UA, and TG in RE treatment were significantly lower (*p* < 0.05). Compared with RE treatment, levels of AST, UA, CHOL, HDL, LDL, and AST/ALT in RR treatment were significantly higher (*p* < 0.05), while those of ALB and TG were significantly lower (*p* < 0.05).

**Table 6:** Effects of RR and RE on serum biochemical parameters of goose.

**Item**	**Group**			***p*-value**
	**Con**	**RR**	**RE**	
ALT (U/L)	12.67 ± 1.53	11.67 ± 0.58	13.67 ± 1.16	0.19
AST (U/L)	32.67 ± 2.08^b^	51.33 ± 0.58^a^	29.33 ± 0.58^c^	1.61 × 10^−6^
ALP (U/L)	961.67 ± 6.43^a^	793.33 ± 10.02^b^	783.67 ± 23.80^b^	1.22 × 10^−6^
AST/ALT	1.55 ± 0.08^c^	4.41 ± 0.28^a^	2.16 ± 0.20^b^	5.67 × 10^−6^
TP (g/L)	54.73 ± 3.61^a^	45.47 ± 0.59^b^	46.90 ± 1.25^b^	5 × 10^−3^
ALB (g/L)	13.4 ± 0.69	12.27 ± 0.15	12.8 ± 1.31	0.34
GLO (g/L)	38.67 ± 0.35^a^	33.20 ± 0.44^c^	34.10 ± 0.44^b^	7.03 × 10^−6^
ALB/GLO	0.35 ± 0.02	0.37 ± 0.01	0.38 ± 0.04	0.38
UA (μmol/L)	297.33 ± 14.36^b^	355.67 ± 2.08^a^	270.33 ± 10.02^c^	1 × 10^−4^
TC (mmol/L)	5.09 ± 0.04^a^	5.20 ± 0.64^a^	4.24 ± 0.32^b^	2 × 10^−3^
TG (mmol/L)	0.93 ± 0.02^b^	0.87 ± 0.01^c^	2.21 ± 0.04^a^	1.62 × 10^−9^
HDL (mmol/L)	2.96 ± 0.05^a^	2.28 ± 0.01^b^	1.87 ± 0.03^c^	4.94 × 10^−8^
LDL (mmol/L)	1.54 ± 0.10^b^	2.31 ± 0.05^a^	1.46 ± 0.12^b^	5.70 × 10^−5^

ALT, alanine aminotransferase; AST, aspartate aminotransferase; ALP, alkaline phosphatase; AST/ALT, aspartate aminotransferase/alanine aminotransferase ratio; TP, total protein; ALB, albumin; GLO, globulin; ALB/GLO, albumin/globulin ratio; UA, uric acid; TC, total cholesterol; TG, triglycerides; HDL, high-density lipoprotein; LDL, low-density lipoprotein.

^a–*c*^Mean values within a row bearing different superscripts differ significantly (p < 0.05).

### 3.6 Immune factors

Compared with Con treatment, RE treatment geese had significantly higher lysozyme levels (*p* < 0.05), and for both RR and RE treatment there was no significant difference in immunoglobulin levels (*p* > 0.05; Table 7).

**Table 7:** Effects of RR and RE on immune factors of goose.

**Item**	**Group**			***p*-value**
	**Con**	**RR**	**RE**	
IgA (g/L)	1.21 ± 0.13	1.41 ± 0.21	1.50 ± 0.22	0.09
IgM (g/L)	0.05 ± 0.008	0.05 ± 0.002	0.05 ± 0.004	0.85
IgG (g/L)	2.23 ± 0.11	2.27 ± 0.17	2.38 ± 0.07	0.20
Lysozyme (μg/mL)	6.97 ± 0.58^b^	7.39 ± 0.27^b^	8.51 ± 0.94^a^	8 × 10^−3^

IgA, immunoglobulin A; IgM, immunoglobulin M; IgG, immunoglobulin G.

^a, b^Mean values within a row bearing different superscripts differ significantly (p < 0.05).

### 3.7 Nutrient digestibility

Compared with Con treatment, Ca-use rates in RR and RE treatment were significantly higher (*p* < 0.05). Addition of RE to goose diet also significantly increased the crude protein use rate (*p* < 0.05; Table 8).

**Table 8:** Effect of RR and RE on nutrient digestibility of goose.

**Item**	**Group**			***p*-value**
	**Con**	**RR**	**RE**	
*P* (%)	52.10 ± 1.81	52.83 ± 0.89	53.25 ± 0.91	0.32
Ca (%)	19.40 ± 0.92^b^	21.14 ± 0.50^a^	21.85 ± 0.51^a^	4.51 × 10^−5^
Crude protein (%)	62.83 ± 0.86^b^	62.48 ± 0.92^b^	64.77 ± 1.09^a^	1.83 × 10^−3^
Total energy (%)	77.69 ± 2.44	77.48 ± 3.30	78.17 ± 3.61	0.93

^a, b^Mean values within a row bearing different superscripts differ significantly (p < 0.05).

### 3.8 Gut microbiota

**Figure 1:**
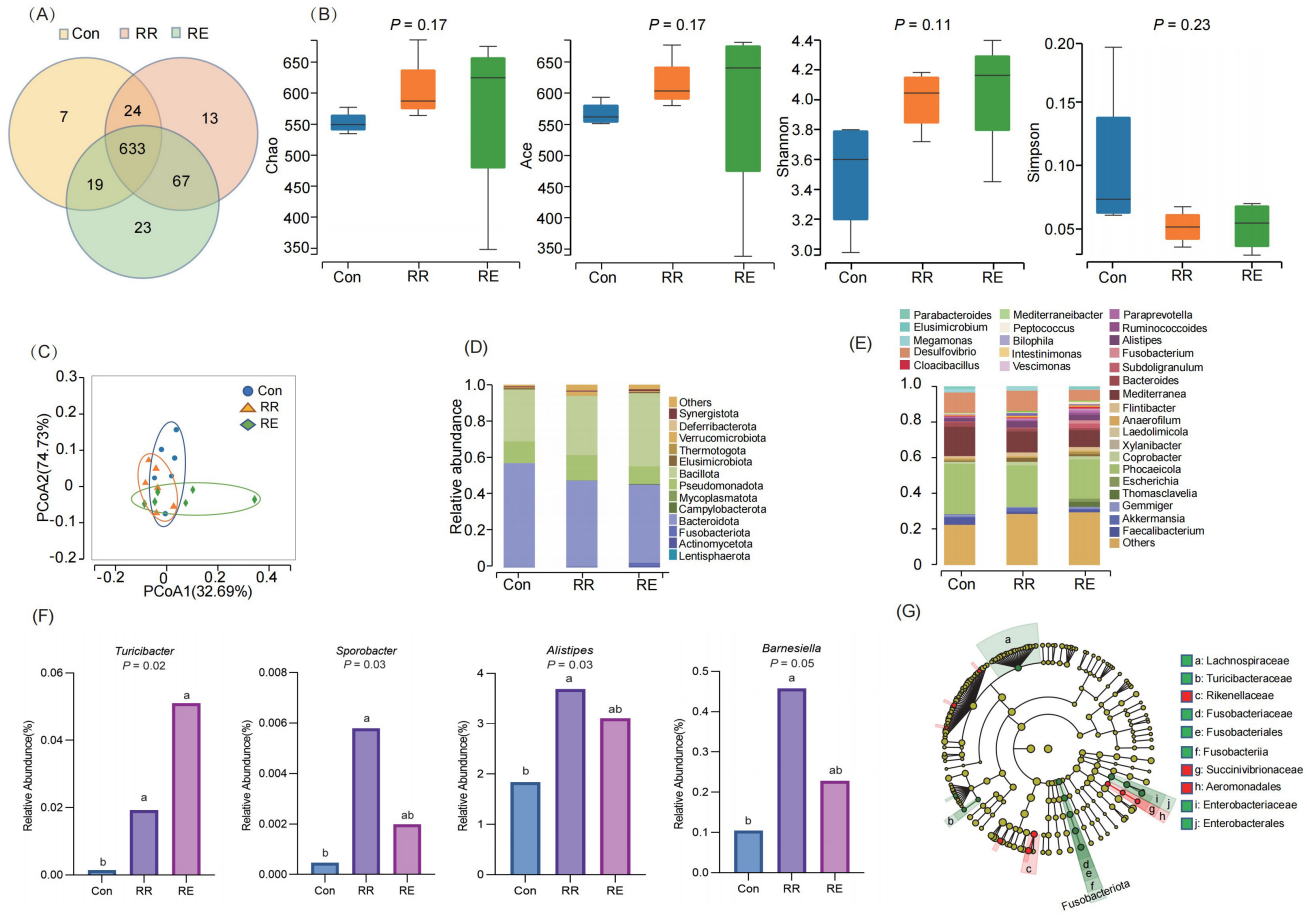
Effects of RR and RE on gut microbiota of goose. **(A)** Venn diagram based on OTU; **(B)** α-diversity analysis; **(C)** PCoA analysis; **(D)** Composition at phylum; **(E)** Composition at genus; **(F)** Difference analysis of genus; Different lowercase letters indicate significant differences among treatments (*p* < 0.05). **(G)** LEfSe analysis.

A total of 790 OTUs were obtained by sequencing (Con 683, RR 737, RE 742), of which 633 were shared among treatments, and 43 were unique (Con 7, RR 13, RE 23; Figure 1A). There were no significant differences among treatments in Chao, ACE, Shannon, and Simpson indices (*p* > 0.05; Figure 1B). Principal coordinates analysis (PCoA) revealed that gut microbial community structures did not differ significantly among treatments, and that community structures overlapped (*r*^2^ = 0.16, *p* = 0.75; Figure 1C). At the level of phylum, communities mainly comprised Bacteroidota (Con 56.69%, RR 46.98%, RE 42.58%), Bacillota (Con 28.35%, RR 32.44%, RE 39.71%), and Pseudomonadota (Con 11.97%, RR 13.85%, RE 9.80%; Figure 1D). At the level of genus, >70% of relative abundance was represented by 28 genera, mainly of *Alistipes, Megamonas, Bacteroides, Faecalibacterium, Desulfovibrio, Mediterranea*, and *Phocaeicola* (Figure 1E). Differential analysis the relative abundance of *Turicibacter* was significantly increased in both the RR and RE treatment compared to the Con treatment (*p* < 0.05), and *Sporobacter, Alistipes*, and *Barnesiella* in RR treatment were also significantly increased than Con treatment (*p* < 0.05; Figure 1F). LEfSe analysis revealed the families Rikenellaceae and Succinivibrionaceae, and Aeromonadales to be enriched in the RR treatment, and for the families Lachnospiraceae, Turicibacteraceae, and Enterobacteriaceae to be enriched in the RE treatment (Figure 1G).

## 4 Discussion

### 4.1 Production performance

Livestock and poultry production performance (e.g., growth, body size, carcass performance) directly affect the economic viability of an industry. We report RE to increase goose ADG and final body weight, which is consistent with studies reporting RE to enhance ADG and carcass weight in chickens (Yesilbag et al., 2011), and significantly improve pre-slaughter weight, ADG, and ADFI of weaned piglets (Yang et al., 2021). Rosemary is rich in active compounds (~22% flavonoids, 12.5% phenols) that through regulation of digestive enzymes and improving intestinal absorption, promote animal growth (Kedir et al., 2023). Flavonoids and saponins promote growth performance by stimulating the pituitary adrenocortical system, prompting release of corticosteroids, enhancing the body's metabolic capacity, and increasing digestion and absorption of nutrients such as proteins and carbohydrates (Milosevic et al., 2018). Adding flavonoids and phenolic acid compounds can enhance ADG, F/G, and ADFI in broiler chickens (Zhou et al., 2019; Fan et al., 2024).

Features of body size indicate animal growth and development, and directly relate to body performance, particularly skeletal development, and carcass performance (Barshan et al., 2019; Guinotte et al., 1991). We report a diet supplemented with RE to increase geese breast depth and neck length. RE contains large concentrations of flavonoids, which stimulate growth and development-related signaling pathways such as activating mitogen-activated protein kinase (MAPK), nuclear factor (NF), Wnt/β-catenin pathway, and bone morphogenetic protein 2 (BMP2)/SMAD. This stimulation promotes proliferation and differentiation of osteoblasts, osteoclast activity, and production of bone matrix, thereby positively influencing bone development, deposition, and reconstruction (Ramesh et al., 2021). Daily consumption of flavonoids as a supplement reduces the decrease in bone density associated with menopausal women (Wong et al., 2009). Supplementing the diet of broiler chickens fed with polyphenolic compounds significantly enhances the carcass yield of broiler chickens, with the degree of improvement increasing at higher supplementation levels (Kousar et al., 2024; Ölmez et al., 2021). Consistent with previous research, we report a diet supplemented with both RR and RE to increase the full and semi-evisceration percentage of geese, possibly because abundant phenolic ingredients in rosemary facilitate nutrient absorption. Polyphenolic ingredients can improve carcass characteristics of broiler chickens by alleviating liver damage and optimizing blood lipid profiles (Karadagoglu et al., 2020). Dietary supplementation with RR did not significantly affect goose growth or body size features, and percentage of full-eviscerated and half-eviscerated compared with RE were also significantly lower, possibly because active ingredients in RR occurred at much lower concentrations than those in RE. This also indicates that carcass traits are more sensitive to the effects of active ingredients in rosemary at low doses. Hence, when evaluating the effects of low dosages of active ingredients on livestock, carcass characteristics should examined.

### 4.2 Organ index

The organ index reflects organ development and functional status. Certain phenolic compounds cause vacuolization of clam digestive organs and damage to membrane structure (Zhang et al., 2024), and in broiler chickens, shorten jejunal villus length and downregulate expression of intestinal tight-junction proteins (Jia et al., 2023). We report geese in both RR and RE treatments to have decreased duodenal indices, and in the RE treatment, for the jejunal index, and the relative lengths of the duodenum and jejunum, to have decreased. This may be related to inhibition of goose digestive organ development by the active ingredients in RR and RE such as flavonoids and polyphenols. These compounds can activate the c-Jun N-terminal kinase and p38-MAPK signaling pathways, promoting release of inflammatory mediators and activation of inflammatory cells, leading to most cells remaining in the S phase (unable to enter G2 phase), causing digestive organ dysplasia (Jia et al., 2021). Flavonoids and aromatic compounds can also activate the AhR/Arnt signaling pathway, causing overexpression of *CYP1A1* enzyme in target organs, which is detrimental to development of digestive organs in zebrafish (Wang et al., 2015). Conversely, a mixed essential oil containing RE increased the weights of the pancreas and small intestine in calves (Campolina et al., 2023), possibly because of differences in animal species and additive.

### 4.3 Serum metabolites

Serum biochemical indicators indicate the body's nutritional and health status. For example, AST and ALP indicate the metabolic capacity of the liver, with elevated levels suggesting potential liver damage (Tamber et al., 2023). A higher AST/ALT ratio signifies more severe liver cell injury (Amernia et al., 2021). We report RE to reduce levels of serum AST and ALP in geese, indicating an improvement in liver metabolic capacity, likely because active ingredients in RE reduced the apoptosis rate and enhanced the body's antioxidant capacity, thereby alleviating liver injury (Ma et al., 2022). Just like the flavonoid compounds can alleviate fatty liver symptoms in obese mice (Park et al., 2023; Toppo et al., 2017). However, we report that RR increased AST levels, possibly because the high fiber content in RR exacerbated heat production in goose, causing chronic heat stress and hindering liver metabolism. In broiler chickens, chronic heat stress increased AST levels and decreased the liver index (Tang et al., 2022). High-dose flavonoid compounds can also alleviate stress and reduce inflammation (Niu et al., 2013), while low-dose flavonoids and phenolic acids can induce activation of FXR–SHP axis, upregulate *IL-6, IL-1*β, and *INF-*γ gene expression, and cause inflammatory liver injury (Zhang G. et al., 2023). Serum TP, GLO, and UA levels reflect the status of protein metabolism in the body (Maiuolo et al., 2016). Supplementation of plant extracts rich in flavonoids and phenolic acids can reduce serum TP and GLO levels in mice by inhibiting liver protein synthesis (Bamikunle et al., 2022), and decrease UA levels in hyperuricemic mice by inhibiting xanthine oxidase activity (Xiang et al., 2022). We report similar changes in serum TP, GLO, and UA levels in geese, although RR increased the UA level, as it did AST, likely because chronic heat stress exacerbated inflammatory responses and inhibited protein synthesis metabolism (Hilsabeck et al., 2022). Chronic heat stress exacerbated protein degradation in Beijing duck, leading to increased UA level (Zeng et al., 2024). Serum LDL, HDL, and TG levels indicate the body's lipid metabolism status. Certain phenolic compounds can hinder fatty acid oxidation in zebrafish, disrupting the lipid metabolism balance (Zhang S. et al., 2022), and some alkaloids and polyphenols can improve the ability of mouse liver to deposit fat (Choi et al., 2017). We report geese in both RE and RR treatments to have variable degrees of abnormal lipid metabolism indicators, possibly because phenolic compounds cause endoplasmic reticulum stress, disrupting the β-oxidation pathway of fatty acids and *PGC1* a-mediated mitochondrial function, which leads to lipid accumulation, or because continuous activation of protein kinase increases expression of *CD36* in hepatocytes, causing increased blood lipids (Choi et al., 2017).

### 4.4 Immune factors

Lysozyme, an important non-specific immune factor that indicates the body's immune status. It functions in sterilization, activating the complement system, phagocytosis, and preventing infectious diseases (Ogundele, 1998). We report increased lysozyme activity in geese in the RE treatment. Rosmarinic acid, rich in RE, can enhance activity of Th2 cytokines and chemokines, reduce the expression of inflammatory cytokines, inhibit phosphorylation of extracellular signal-regulated kinases, c-Jun N-terminal kinase, and p38, and effectively delay the inflammatory response (Liang et al., 2016; Estaiano de Rezende et al., 2021). Additionally, flavonoid compounds can reduce secretion of IL-4, IL-5, and IL-13 from immune cells *in vivo*, and increase mRNA expression of *AMCase, CCL11, CCR3, YM2*, and *E-selectin*, thereby inhibiting the inflammatory process (Liang et al., 2016). Adding flavonoids can also increase lysozyme levels in Nile tilapia and mice (Estaiano de Rezende et al., 2021; Gao et al., 2017).

### 4.5 Nutrient digestibility

Nutrient digestibility, an important factor that affects production performance of livestock and poultry (Tamber et al., 2023), measures the body's digestion and nutrient use. For weaned piglets, RE increased the height and ratio of villi in the jejunum and ileum, improved nutrient digestion and absorption, and increased the digestibility of crude protein and total energy (Yang et al., 2021). For geese, we report both RE and RR to improve Ca absorption, and for RE to also enhance crude protein use. Flavonoid and phenolic acid compounds in rosemary can improve intestinal morphology, and their involvement in inhibiting deamination and dehydrogenation processes during bacterial proliferation in *vivo*, and enhance the ability of the body to use nutrients (Lin et al., 2022). Pre-culturing cellulase with various phenolic acid compounds changes the cellulase polymerization reaction, causing -COOH and -CH_3_O groups to interact with hydrophobic groups and improve cellulase activity (Ran et al., 2022). This enhanced cellulase activity can inhibit degradation of crude protein by some gut spoilage microbiota by providing substrates for lactic acid bacteria (Zhang et al., 2019). For carp, adding cellulase to the diet increased the apparent digestibility of crude protein, crude fat, and carbohydrate (Dawood and Shi, 2022).

### 4.6 Gut microbiota

The host's diet and immune status have a strong selective effect on intestinal microbiota, while intestinal microbiota can sense changes in the host's intestine, and affect host metabolism by adjusting their own structure and gene expression (Cani, 2016). For geese, we report that supplementing the diet with rosemary did not significantly affect gut microbiota α or β diversity, indicating that gut microbiota stability enabled normal growth and development. Bacteroidota, Bacillota, and Pseudomonadota were dominant phyla in geese guts, accounting for ~95% of all phyla, similar to results reported by Gao et al. (2016). We found that the relative abundance of the genus *Turicibacter* increased significantly in the RE group, which was further supported by LEfSe analysis that the family Turcibacteraceae to be enriched in the RE treatment. This suggests that *Turicibacter* and its affiliated family could be pivotal in shaping the functionality of the microbial community, and consequently, the health of the host. *Turicibacter*, through the production of bile salt hydrolase and 7α-hydroxysteroid dehydrogenase, can modulate the host's bile acid metabolism, leading to a reduction in serum cholesterol and TG level, and adipose tissue content, and consequently improving the lipid metabolism (Lynch et al., 2023). Meanwhile, with the increase of the abundance of *Turicibacter* in the gut of hypercholesterolemic rats, the TC level was reduced and the gut metabolic spectrum was improved (Huang et al., 2021). In addition, *Sporobacter, Alistipes* and *Barnesiella* were also significantly increased in RR group, which may be because the polyphenol compounds contained in rosemary. Firstly, increase the levels of short chain fatty acids, especially acetic acid and propionic acid, providing more energy for gut microbiota. Secondly, reduce the inflammatory response, providing more favorable conditions for the growth of gut beneficial bacteria (Schytz Andersen-Civil et al., 2024).

Furthermore, families Rikenellaceae and Succinivibrionaceae, and Aeromonadales to be enriched in the RR treatment, and for the families Lachnospiraceae, Turicibacteraceae, and Enterobacteriaceae to be enriched in the RE treatment. Phenolic acids increased the abundance of Rikenellaceae in the gut, and suppressed gut inflammation and protected host cells from oxidative stress and improved gut epithelial barrier functions (Dong et al., 2022; Jones-Hall et al., 2015). Succinivibrionaceae benefited the host in various ways (e.g., as anti-inflammatory effects, and maintaining gut integrity; Lin et al., 2021), and their abundance in mice after recovery from kidney disease was significantly higher than it was in mice that had died, indicating that they promote growth and maintain health (Zhang L. et al., 2022). Aeromonadales is associated with reduced systemic inflammation, and its increased abundance in the gut of mice with chronic kidney disease slowed down renal fibrosis (Zhang S. et al., 2022). In general, RR is mainly involved in the maintenance of host gut barrier functions, and it improves body health by enriching microbiota with anti-inflammatory effects. Lachnospiraceae in the gut can convert primary bile acids into secondary bile acids and produce short-chain fatty acids, butyrate salts, and some peptide antibiotics that inhibit pathogenic bacteria. Additionally, because of its enrichment near the mucosa, it is extensively involved in epithelial and mucosal immunity (Berger et al., 2021). An increased abundance of Lachnospiraceae in the gut of broiler chickens led to significant increases in production of butyrate, which significantly improved body weight (Yacoubi et al., 2018). Turicibacteraceae metabolizes to produce a large amount of butyric, acetic, and valeric acids, and other short-chain fatty acids, which play an important role in regulating gut health (Gao et al., 2021; Liu et al., 2021). Polyphenols increased the abundance of gut Turicibacteraceae in mice, reducing endotoxemia markers, and improving immunity (Zhang et al., 2017). Enterobacteriaceae play an important role in anti-cancer, anti-inflammatory, anti-mutation, anti-allergy, and neuroprotective effects (Burapan et al., 2017), and its abundance in gut of kidney transplant patients correlates negatively with the probability of urinary tract infection (Magruder et al., 2020). Overall, RE mainly maintains body health levels and promotes growth by enriching microbiota that enhance host immunity.

## 5 Conclusion

Supplementing the diet of geese with 0.2% RE comprehensively improve production, immune performance, and composition of gut microbiota, while 15% RR only improve production and composition of gut microbiota to a certain extent. However, because RR is available in greater quantity and at a lower price than RE, and is otherwise a waste product, its use in animal production is warranted, and using it, a significant waste problem associated with extraction of active ingredients from rosemary is also resolved. Therefore, it represents a potentially viable green additive to improve animal production.

## Data Availability

The original contributions presented in the study are publicly available. This data can be found in the NCBI's Sequence Read Archive with the accession number PRJNA1199177.
