# Corrigendum: Effects of rosemary extract and its residue on production, immune performance, and gut microbiota in geese

**DOI:** 10.3389/fmicb.2025.1561085

**Published:** 2025-01-28

**Authors:** Yuzhi Huang, Lanmeng Xu, Hang He, Lijuan Peng, Qinfeng Liao, Kun Wan, Simeng Qin, Lijing Cao, Jie Zhang

**Affiliations:** ^1^College of Animal Science and Technology, Southwest University, Chongqing, China; ^2^College of Animal Science and Technology, Chongqing Three Gorges Vocational College, Chongqing, China; ^3^Chongqing Rongchang District Vocational Education Center, Chongqing, China

**Keywords:** rosemary extract, rosemary extract residue, production performance, immune performance, gut microbiota, geese

In the published article, there was an error in Table 1 as published. The composition of basal diet was incomplete as it was missing the ingredient “Rice bran” with a content of 13.40%. The corrected Table 1 appear below.

**Table 1:** Composition and nutrient level of basal diet (air-dry basis).

**Ingredients**	**Content (%)**	**Nutrient**	**Content**
Corn	53.60	Metabolizable energy (MJ/kg)^b^	11.21
Soybean meal	11.50	Crude protein (%)	14.81
Wheat bran	14.50	Crude fiber (%)	8.04
Rice bran	13.40	Calcium (%)	0.80
Silkworm chrysalis	1.79	Available P (%)^b^	0.40
CaHPO_4_	0.90	Lysine (%)	0.85
Salt (NaCl)	0.20	Methionine (%)	0.30
Limestone	0.75		
L-Lysine (98%)	0.18		
DL-Methionine	0.07		
Choline chloride	0.12		
Premix^a^	2.00		
Sand	1.00		
Total	100		

^a^Premix contained the following per kg of diet: VA 40,000 IU, VD_3_ 2,000 IU, VE 60 mg, VK_3_ 2 mg, VB_1_ 4 mg, VB_2_ 24 mg, VB_6_ 4 mg, VB_12_ 50 μg, nicotinic acid 12 mg, pantothenic acid 36 mg, folic acid 4 mg, biotin 0.4 mg, Fe 120 mg, Cu 4 mg, Mn 300 mg, Zn 160 mg, I 0.2 mg, and Se 0.2 mg.

^b^ME was a calculated value, while the others were measured values.

The authors apologize for this error and state that this does not change the scientific conclusions of the article in any way. The original article has been updated.

